# Enhanced diagnostic accuracy for quantitative bone scan using an artificial neural network system: a Japanese multi-center database project

**DOI:** 10.1186/2191-219X-3-83

**Published:** 2013-12-26

**Authors:** Kenichi Nakajima, Yasuo Nakajima, Hiroyuki Horikoshi, Munehisa Ueno, Hiroshi Wakabayashi, Tohru Shiga, Mana Yoshimura, Eiji Ohtake, Yoshifumi Sugawara, Hideyasu Matsuyama, Lars Edenbrandt

**Affiliations:** 1 Department of Nuclear Medicine, Kanazawa University Hospital, 13-1 Takara-machi, Kanazawa 920-8641, Japan; 2 Department of Radiology, St. Marianna University School of Medicine, Kawasaki 216-8511, Japan; 3 Department of Diagnostic Radiology, Gunma Prefectural Cancer Center, Ota 373-8550, Japan; 4 Department of Urology, International Medical Centre-Comprehensive Cancer Centre, Saitama Medical University, Kawagoe 350-1298, Japan; 5 Department of Nuclear Medicine, Hokkaido University Graduate School of Medicine, Sapporo 060-8638, Japan; 6 Department of Radiology, Tokyo Medical University, Tokyo 160-8402, Japan; 7 Department of Nuclear Medicine, Kanagawa Cancer Center, Yokohama 241-8515, Japan; 8 Department of Diagnostic Radiology, Shikoku Cancer Center, Matsuyama 791-0280, Japan; 9 Department of Urology, Yamaguchi University School of Medicine, Yamaguchi 755-8505, Japan; 10 Department of Clinical Physiology and Nuclear Medicine, Gothenburg University, Gothenburg 41345, Sweden

**Keywords:** Bone scintigraphy, Bone scan index, Artificial neural network, Databases, Multi-center study

## Abstract

**Background:**

Artificial neural network (ANN)-based bone scan index (BSI), a marker of the amount of bone metastasis, has been shown to enhance diagnostic accuracy and reproducibility but is potentially affected by training databases. The aims of this study were to revise the software using a large number of Japanese databases and to validate its diagnostic accuracy compared with the original Swedish training database.

**Methods:**

The BSI was calculated with EXINIbone (EB; EXINI Diagnostics) using the Swedish training database (*n* = 789). The software using Japanese training databases from a single institution (BONENAVI version 1, BN1, *n* = 904) and the revised version from nine institutions (version 2, BN2, *n* = 1,532) were compared. The diagnostic accuracy was validated with another 503 multi-center bone scans including patients with prostate (*n* = 207), breast (*n* = 166), and other cancer types. The ANN value (probability of abnormality) and BSI were calculated. Receiver operating characteristic (ROC) and net reclassification improvement (NRI) analyses were performed.

**Results:**

The ROC analysis based on the ANN value showed significant improvement from EB to BN1 and BN2. In men (*n* = 296), the area under the curve (AUC) was 0.877 for EB, 0.912 for BN1 (*p* = not significant (ns) vs. EB) and 0.934 for BN2 (*p* = 0.007 vs. EB). In women (*n* = 207), the AUC was 0.831 for EB, 0.910 for BN1 (*p* = 0.016 vs. EB), and 0.932 for BN2 (*p* < 0.0001 vs. EB). The optimum sensitivity and specificity based on BN2 was 90% and 84% for men and 93% and 85% for women. In patients with prostate cancer, the AUC was equally high with EB, BN1, and BN2 (0.939, 0.949, and 0.957, *p* = ns). In patients with breast cancer, the AUC was improved from EB (0.847) to BN1 (0.910, *p* = ns) and BN2 (0.924, *p* = 0.039). The NRI using ANN between EB and BN1 was 17.7% (*p* = 0.0042), and that between EB and BN2 was 29.6% (*p* < 0.0001). With respect to BSI, the NRI analysis showed downward reclassification with total NRI of 31.9% ( *p* < 0.0001).

**Conclusion:**

In the software for calculating BSI, the multi-institutional database significantly improved identification of bone metastasis compared with the original database, indicating the importance of a sufficient number of training databases including various types of cancers.

## Background

Bone scintigraphy has been accepted as a means to identify bone metastases associated with various types of cancer. Even after the advent of single-photon emission computed tomography combined with X-ray computed tomography, whole-body bone imaging is a standard method to survey the existence and extent of bone metastasis. Moreover, although bone scan interpretation may be performed on visual interpretation of whole-body images, an appropriate quantitative approach has been expected. While initial detection of bone metastases is important, quantification of progress of metastasis that results in patients’ disability, pain, pathological fractures, and mortality would be also beneficial [1,2]. However, there had been no definite imaging method that reflected metastatic disease burden and treatment effect before the advent of bone scan index (BSI) proposed at Memorial Sloan-Kettering Cancer Center [3].

The BSI was developed as a marker of the spread for bone metastasis, which is a fraction of bones involved by a tumor and which realizes the regional distribution of the lesions [4]. The software program for calculating BSI using the neural network system has also been developed using whole-body images with a Swedish database [5]. They successfully applied automatic segmentation of the skeletal regions and automatic detection and feature extraction of hot spots using the neural network system. However, the diagnostic accuracy is potentially influenced by training databases. Whether the same database can be used universally in any study population is yet to be determined. The initial version using a Japanese database showed promising results with a revised database, but it was based on a single-center database [6,7].

The aims of this study were to create a multi-center Japanese database based on a large number of subjects with and without definite bone metastasis and to test the diagnostic accuracy compared with the original European database. In addition, to understand the characteristics of diagnostic accuracy based on the new database, a net reclassification improvement analysis was performed [8].

## Methods

### Patients

The new multi-center training database used in the development of BONENAVI version 2 (BN2) comprised 1,532 patients from nine Japanese hospitals (Table 1). The average age was 68 ± 10 (range 20 to 99) years for males and 59 ± 12 (range 26 to 91) years for females. A total of 42% of the patients had bone metastasis, with the underlying cancer being prostate cancer in 29%, breast cancer in 41%, and other cancers in 30% of the cases. In all hospitals, radiology and/or nuclear medicine specialists made the definitive diagnoses. Every hot spot was classified as metastasis or not, based on information from multiple modalities including X-ray computed tomography (CT), magnetic resonance imaging, positron emission computed tomography, and serial bone scan follow-up studies. All patients had X-ray CT studies, and 1,434 (94%) patients had two or more bone scans.

**Table 1:** Demographics of European and Japanese databases and validation groups

	**EB**	**BN1**	**BN2**	**Validation group**		
				**All**	**Male**	**Female**
*N*	789	904	1,532	503	296	207
Age (years)	66 ± 12	64 ± 12	64 ±12	65 ± 11	68 ±9	59 ± 12
Male, *N* (%)	508 (64)	457 (51)	790 (52)	296 (59)	-	-
Bone metastases, *N* (%)	262 (33)	141 (16)	638 (42)	169 (34)	96 (32)	73 (35)
Types of cancer						
Prostate, *N* (%)	425 (54)	267 (30)	451 (29)	207 (41)	207 (70)	-
Breast, *N* (%)	217 (28)	383 (42)	624 (41)	166 (33)	-	166 (80)
Others, *N* (%)	147 (19)	254 (28)	457 (30)	130 (26)	89 (30)	41 (20)

EB, EXINIbone; BN1, BONENAVI version 1; BN2, BONENAVI version 2.

Blood sampling including biochemical bone and tumor markers and the patient clinical courses were also used to reach the gold standard classification. Hot spots most likely due to degenerative disease or arthritis, for example, in the vertebrae and shoulder joints, were judged as non-metastatic.

The performance of BN2 was compared to that of EXINIbone (EB; version 1.3, EXINI Diagnostics AB, Lund, Sweden) and the first version of BONENAVI (BN1; collaboration between EXINI Diagnostics AB and FUJIFILM RI Pharma, Co. Ltd, Tokyo, Japan). The training database of EB comprised 789 bone scans from a single Swedish hospital [9], and that of BN1 comprised 904 bone scans from a single Japanese hospital [7]. The demographics of the patients from the different training databases are shown in Table 1.

A validation group, used to test the performance of EB, BN1, and BN2, was developed as a second multi-center group of 503 patients from the same nine Japanese hospitals that participated in the BN2 training database (Table 1). The classification criteria regarding metastatic diseases were the same as in BN2. The underlying malignancies other than breast cancer and prostate cancer (*n* = 130) were lung cancer (*n* = 49), renal cancer (*n* = 17), esophageal cancer (*n* = 8), gastric cancer (*n* = 6), thyroid cancer (*n* = 5), pancreatic cancer (*n* = 5), and other types of malignancy (*n* = 4 or less for each type).

To accumulate the scintigraphic images for the databases, even though the DICOM data were anonymized, approval of the institutional review board or ethical committee was obtained in all institutions. All the data were accumulated retrospectively. The review boards waived the written informed consent from each patient.

### Whole-body bone scan

Whole-body anterior and posterior images were used for the analysis. A standard dose of 555 to 740 MBq of ^99m^Tc-methylene diphosphonate (MDP; FUJIFILM RI Pharma, Co. Ltd, Tokyo, Japan) was injected and imaged 3 h (range 2.5 to 5.5 h) later. The matrix size was 256 × 1,024. Energy peak was centered at 140 keV with 15% to 20% windows.

### Automated bone scan analysis

The automated method for analysis of anterior and posterior whole-body bone scan images has been described previously [9]. Segmentation of the skeleton was performed by fitting an atlas to the patient skeleton using Morphon registration for non-rigid image registration. The atlases were based on 10 normal bone scans from European patients for EB and 23 normal bone scans from Japanese patients for BN1, whereas gender-specific atlases were developed for BN2 using normal bone scans from 25 male and 25 female Japanese patients. Regions inside the delineated skeleton with intensities exceeding a threshold were defined as hot spots. This threshold varied over different parts of the skeleton and was proportional to the overall intensities found in a neighborhood surrounding each hot spot. This made the algorithm equally sensitive to hot spots in low-intensity regions such as the ribs and high-intensity regions such as the lumbar spine. Each individual hot spot was classified as metastasis or not by an artificial neural network (ANN). Separate ANNs were used for each anatomical region, e.g., skull, spine, ribs, pelvis, and femur, and the different ANNs were used as input for specific sets of variables such as size, shape, intensity, and localization of the hot spot. The training databases for EB, BN1, and BN2 were different as described above, and gender-specific ANNs were developed for BN2. The training of the ANNs was performed using customized software at EXINI Diagnostics. The skeletal involvement of each hot spot was calculated as the percentage of the total skeleton, and the BSI was calculated as the sum of the skeletal involvement of all hot spots classified as metastases by the ANNs.

### Statistical analysis

All the data were expressed as an average and standard deviation. Contingency table analysis was performed to compare values in two groups. The receiver operating characteristic (ROC) analysis was performed and the area under the curve (AUC) was calculated. An optimal cutoff for the sensitivity and specificity could be calculated as the highest value of sensitivity − (1 − specificity). The net reclassification improvement (NRI) analysis for identifying bone metastasis was performed using four ANN groups of 0 to 0.24, 0.25 to 0.49, 0.50 to 0.74, and 0.75 to 1.00 [8]. In order to evaluate the effect of the software revisions on BSI, the NRI analysis was also performed using four BSI groups of <0.1, 0.1 to 0.99, 1 to 4.99, and ≥5. *P* values <5% were considered significant.

## Results

### ROC and optimal sensitivity and specificity

**Figure 1:**
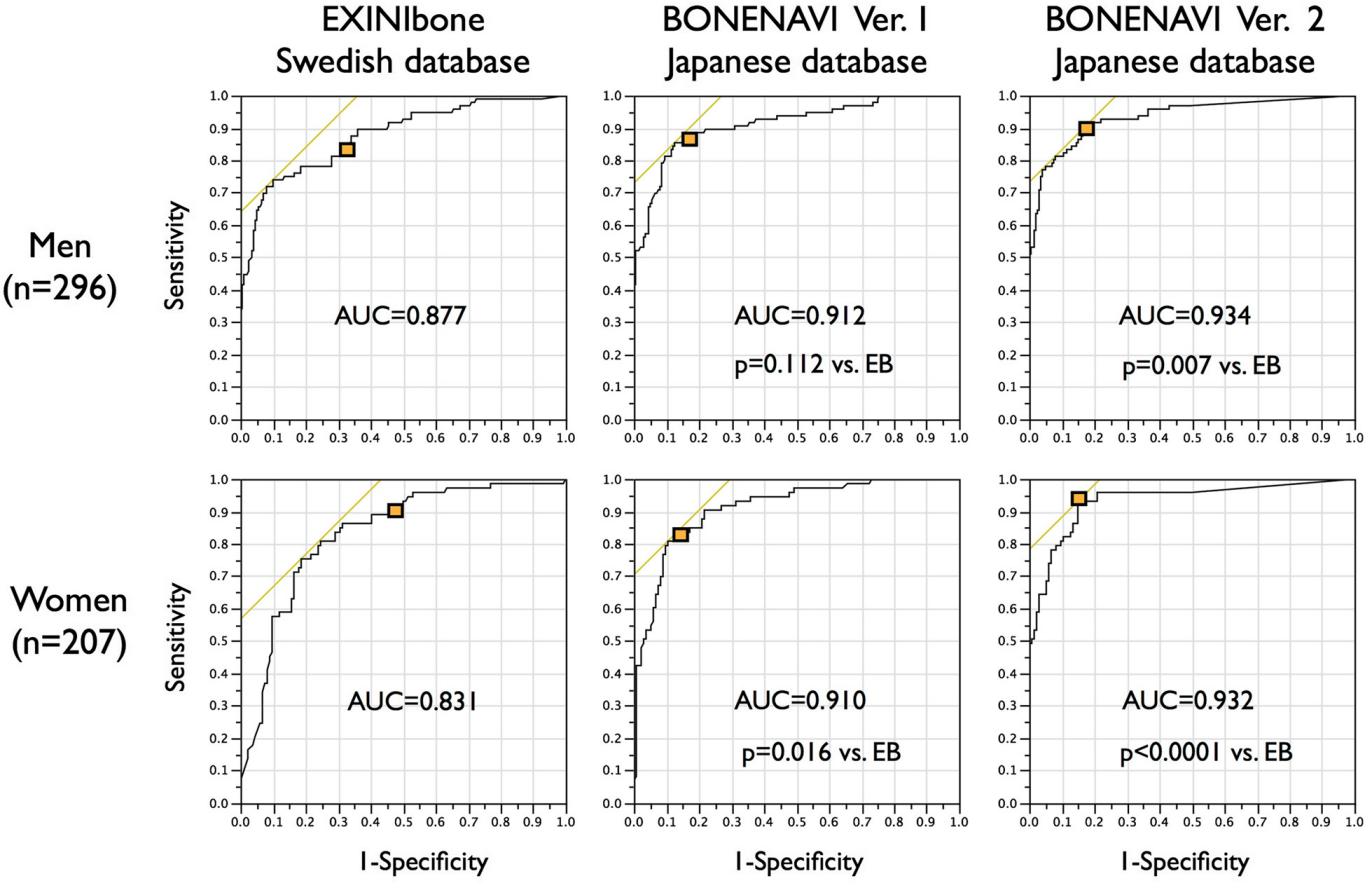
**Diagnostic accuracy based on ANN assessed by ROC analysis for EB, BN1, and BN2.** Squares in graphs indicate sensitivity and specificity adjusted for optimal balance of ANN, while tangential lines indicate the highest sensitivity − (1 − specificity).

Diagnostic accuracy based on ANN was examined using ROC analysis (Figure 1). Since the databases were made separately for men and women, the ROC AUC based on ANN was calculated in each gender. In men (*n* = 296), the AUC was 0.877, 0.912, and 0.934 for EB, BN1 (*p* = not significant (ns) vs. EB), and BN2 (*p* = 0.007 vs. EB), respectively. The sensitivity was determined as 83% for EB, 88% for BN1 (*p* = ns vs. EB), and 90% for BN2 (*p* = ns vs. EB), whereas specificity was determined as 69% for EB, 83% for BN1 (*p* = 0.001 vs. EB), and 84% for BN2 (*p* = 0.001 vs. EB). In women (*n* = 207), the AUC was 0.831, 0.910, and 0.932 for EB, BN1 (*p* = 0.016 vs. EB), and BN2 (*p* < 0.001 vs. EB), respectively. The sensitivity was determined as 90% for EB, 81% for BN1 (*p* = 0.167 vs. EB), and 93% for BN2 (*p* = 0.774 vs. EB), whereas the specificity was determined as 51% for EB, 87% for BN1 (*p* < 0.001 vs. EB), and 85% for BN2 (*p* < 0.001 vs. EB).

**Figure 2:**
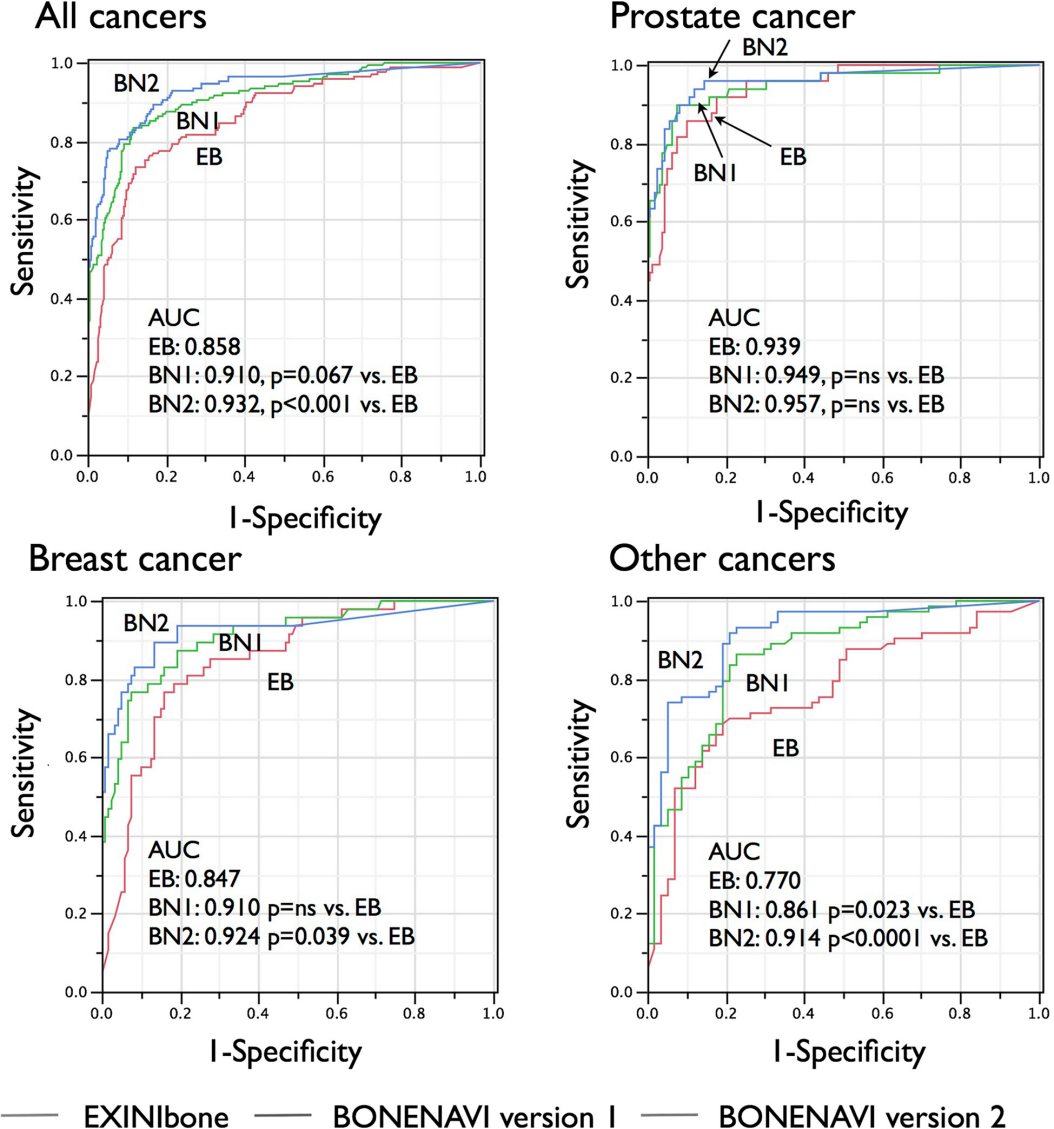
**Diagnostic accuracy based on ANN evaluated by ROC analysis for EB (red), BN1 (green), and BN2 (blue).** The ROCs are compared in the groups of prostate cancer, breast cancer, and other cancers.

Figure 2 shows differences in ROC curves depending on cancer types. When ANN was used to discriminate metastatic patients with both genders combined, the ROC AUC was 0.858 for EB, 0.910 for BN1 (*p* = 0.067 vs. EB), and 0.932 for BN2 (*p* < 0.0001 vs. EB). In patients with prostate cancer, the ROC AUC was not improved from EB (0.939), BN1 (0.949), to BN2 (0.957). In patients with breast cancer, however, AUC was improved from EB (0.847) to BN1 (0.910, *p* = ns) and EB to BN2 (0.924, *p* = 0.039). In patients with other cancers, AUC was significantly improved from EB (0.770) to BN1 (0.861, *p* = 0.023) and EB to BN2 (0.914, *p* < 0.0001).

When NRI analys is was performed between EB and BN2, net gain in reclassification proportion in patients with metastasis (*n* = 169) was 3.6% (*p* = ns), whereas it was −26.0% (*p* < 0.0001) in patients without metastasis (*n* = 334) (Table 2). Total NRI was 29.6% and was highly significant (*p* < 0.0001). The NRI from EB to BN1 was 17.7% (*p* = 0.0042) with net gains of −5.3% (*p* = ns) and −23.1% (*p* < 0.0001) in patients with and without metastasis, respectively. The NRI from BN1 to BN2 was 10.4% (*p* = 0.064) with net gains of 10.1% (*p* = 0.020) and −0.3% (*p* = ns) in patients with and without metastasis, respectively.

**Table 2:** Net reclassification improvement analyses between EB and BN2 based on ANN groups

	**EB**	**BN2**				
		**0 to 0.24**	**0.25 to 0.49**	**0.50 to 0.74**	**0.75 to 1.00**	**Total**
Metastasis (*n* = 169): net gain in reclassification proportion = 3.6%, *p* = 0.38	0 to 0.24	1	3	3	3	10
		0.6%	1.8%	1.8%	1.8%	
	0.25 to 0.49	2	2	4	5	13
		1.2%	1.2%	2.4%	3.0%	
	0.50 to 0.74	2	1	5	8	16
		1.2%	0.6%	3.0%	4.7%	
	0.75 to 1.00	1	3	11	115	130
		0.6%	1.8%	6.5%	68.0%	
	Total	6	9	23	131	169
No metastasis (*n* = 334): net gain in reclassification proportion = −26.0%, *p* < 0.0001	0 to 0.24	96	31	14	4	145
		28.7%	9.3%	4.2%	1.2%	
	0.25 to 0.49	41	14	5	2	62
		12.3%	4.2%	1.5%	0.6%	
	0.50 to 0.74	34	19	9	5	67
		10.2%	5.7%	2.7%	1.5%	
	0.75 to 1.00	35	11	8	6	60
		10.5%	3.3%	2.4%	1.8%	
	Total	206	75	36	17	334

Total NRI = 29.6%, *p* < 0.0001.

NRI analysis was also performed to evaluate the effect of revision on BSI (Table 3). When EB and BN2 were compared in patients with metastasis, the net gain in reclassification proportion in patients with metastasis was −40.8% (*p* < 0.0001). In patients without metastasis, the net gain was −72.8% (*p* < 0.0001). The total NRI was 31.9% and was highly significant (*p* < 0.0001).

**Table 3:** Net reclassification improvement analyses between EB and BN2 based on BSI groups

	** EB**	**BN2**				
		**<0.1**	**0.1 to 1**	**1 to 5**	**>5**	**Total**
Metastasis (*n* = 169): net gain in reclassification proportion = −40.8%, *p* < 0.0001	<0.1	3	0	0	0	3
		1.8%	0.0%	0.0%	0.0%	
	0.1 to 1	6	28	0	0	34
		3.6%	16.6%	0.0%	0.0%	
	1 to 5	4	48	40	0	92
		2.4%	28.4%	23.7%	0.0%	
	>5	0	1	10	29	40
		0.0%	0.6%	5.9%	17.2%	
	Total	13	77	50	29	169
No metastasis (*n* = 334) net gain in reclassification proportion = −72.8%, *p* < 0.0001	<0.1	43	4	0	0	47
		25.4%	2.4%	0.0%	0.0%	
	0.1 to 1	129	31	0	0	160
		76.3%	18.3%	0.0%	0.0%	
	1 to 5	63	38	9	0	110
		37.3%	22.5%	5.3%	0.0%	
	>5	7	7	3	0	17
		4.1%	4.1%	1.8%	0.0%	
	Total	242	80	12	0	334

Total NRI = 31.9%, *p* < 0.0001.

**Figure 3:**
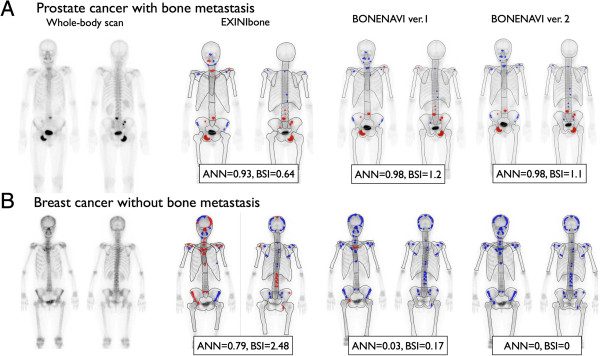
**A 69-year-old man with prostate cancer (A) and a 53-year-old woman with breast cancer (B).** The patient with prostate cancer had multiple metastases that were correctly identified by BN2, and the BSI was increased with BN2 compared with EB. The patient with breast cancer did not have metastasis, and both ANN and BSI were reduced by the revised versions with Japanese training databases. Red hot spots denote high-risk lesions, namely high probability of metastases, whereas blue hot spots denote low-risk lesions.

Figure 3 shows a patient with prostate cancer with bone metastasis and a patient with breast cancer without bone metastasis. In the patient with prostate cancer, the metastatic lesions were correctly identified by BN2. The breast cancer patient showed a high BSI with EB and a lower BSI in BN1. The BSI was correctly diagnosed as 0 with BN2.

## Discussion

This study was performed as a multi-center project to establish a software program by incorporating a database that includes large number of patients with bone metastasis from various cancer types. While the software based on a Japanese single-center database improved the diagnostic accuracy compared with the software based on the original European database, the multi-center database including 1,532 patients further enhanced the diagnostic accuracy. The large training database also made it possible to use gender-specific analysis in BN2.

In addition to the diagnostic use of the software, BSI provides a quantitative measure that reflects the tumor burden expressed as a percentage of total body skeletal mass. The initial study started at Memorial Sloan-Kettering Cancer Center in patients with prostate cancer and showed good reproducibility and a parallel change with prostate-specific antigen [3,4]. BSI has been proved to contain prognostic information in addition to that of conventional prognostic markers such as clinical T stage, Gleason score, and prostate-specific antigen, and it has therefore drawn the attention of oncologists and urologists [10]. When prostate cancer patients were stratified into, for example, high, intermediate, and low BSI groups, significant differences in survival rate were demonstrated [11-13]. On-treatment changes in BSI could be a good response indicator rather than prostate-specific antigen alone in patients with castration-resistant metastatic prostate cancer.

The quantification of bone scans became practical by the use of a computer-aided diagnosis system with ANN, since the quality of visual bone scan interpretation varied according to readers’ experiences [14]. When the segmentation of the skeletons, hot spot detection, evaluation of the characteristics of hot areas, and summed quantitative indexes were available with an automatic method, the reproducibility could be enhanced [5,15]. In a study using EB, a close correlation was demonstrated between manual and automated BSI measurements, and the merit of the latter was 100% reproducibility [9]. Owing to simple application, BSI could be incorporated into clinical practice, while patients were diagnosed, treated, and followed up on.

Training databases are essential for a neural network system to diagnose bone metastases. In this study, we used only Japanese patients with definite diagnosis for the existence of bone metastasis. In addition to patient-based diagnostic accuracy, all the hot areas were confirmed by other imaging modalities and/or follow-up bone scans. Since the BN1 included only 141 (16%) patients with bone metastases from one hospital, it was increased to 638 (42%) patients from nine hospitals. The number of hot spots in ribs, for example, was increased from 2,303 (metastasis 50%) in BN1 to 3,294 (metastasis 43%) in BN2, which contributed to enhancing the learning volumes. When it is utilized in a number of hospitals, the multi-center database judged by multiple experts would be beneficial for enhancing diagnostic accuracy in computer learning.

The larger collection of databases including various cancer types is essential for obtaining appropriate BSI values. When we used the EB on Japanese patients for the first time, hot spots indicating high probability of abnormality were frequently noticed in the skull, shoulder joints, and lumbar vertebrae. These regions included diffuse metabolic accumulation in the skull of female patients and degenerative changes in the vertebrae and joints. About half (*n* = 425) of the Swedish database was from prostate cancer and 28% (*n* = 217) from breast cancer. In contrast, the Japanese databases for BN2 included 29% (*n* = 451) from prostate cancer, 41% (*n* = 624) from breast cancer, and 30% (*n* = 457) from other cancer types. The BN2 databases, therefore, included various cancer types and were closer to the usual clinical environment. From the viewpoint of Japanese population-specific databases, not only the physical stature but also the incidence of degenerative or deformative bone changes might differ between Swedish and Japanese subjects. When EB and BN1 were compared, NRI analysis with ANN showed that BN1 increased negative cases in patients without metastasis, indicating significantly decreased false-positive cases. BN2 further adjusted the diagnostic accuracy and reclassified the metastatic lesions into the higher ANN groups. With respect to the influence of revisions on BSI, the NRI analysis showed that reclassification was downward in both metastatic and non-metastatic groups. However, reclassification of non-metastatic patients into the lower risk BSI seemed to have meaning, and total net reclassification was improved in one third of the patients. The final effect of revision on predicting prognosis should be confirmed in future follow-up studies.

Notable effects of training databases differ among prostate, breast, and other cancers. The differences among cancer types seemed to be related to osteoblastic and osteolytic activity of the bone metastases and their imbalance in regulation [16]. Quantitative measurement of bone metastasis or BSI has most widely been used in patients with prostate cancer [9,10,12,17,18]. Prostate cancer shows typical osteoblastic metastasis based on radiological findings, though it is also associated with osteoclastic process and bone resorption. The bone scan appearance in prostate cancer reveals multiple hot spots and even the so-called superscan in extreme situations. Detecting all metastatic hot areas is important when demanding an overview of the whole amount of metastasis in prostate cancer. Therefore, the diagnostic accuracy in identifying bone metastasis was high even with EB, and further improvement by BN1 and BN2 was not achieved. In contrast, breast cancer commonly metastasizes to bones and destroys its structure, which causes both osteolytic and osteoblastic appearance in bones. The bone scan might show relatively mild activity or even cold areas in the pure osteolytic lesions. Higher fractions of breast and other cancer types in BN2 as compared with EB were also noted, namely, non-prostate cancer, 46% (*n* = 364) for EB, 70% (*n* = 637) for BN1, and 71% (*n* = 1081) for BN2 (Table 1). To enhance the diagnostic accuracy in breast cancer metastasis, decreasing false-positive hot spots had practically important meaning, and it explained why the diagnostic improvement was obtained in BN1 and BN2 as compared with EB.

### Limitations

The detection of metastasis was based on the hot areas, and cold lesions were not included for training the ANN system. However, since most of the diagnosis of the bone metastasis was made by the accumulation of ^99m^Tc-MDP, the utility of BSI would not be substantially changed. Although database training was performed using all subjects, specific cancer type-based training, for example, prostate cancer-specific and breast cancer-specific training databases, could be applied. This process requires considerable time for separate training and will be studied in future works. Finally, when the NRI analysis is performed based on skeletal-related events, instead of diagnosis of metastases, true values of BSI will be confirmed in the future.

Even when we consider the possibility of ^18^ F NaF positron emission computed tomography in the future, a similar approach using ANN and new training databases might be an interesting project. What kinds of algorithm of ANN system are appropriate for tomographic images and/or maximum intensity projection images should be investigated.

## Conclusion

The Japanese multi-center database significantly improved the diagnostic accuracy, showing AUC of 0.93 in both genders with ANN. The improvement from EB to BN1 to BN2 was particularly high in patients with breast cancer and other cancer types, while the diagnostic accuracy was equally high in patients with prostate cancer. Reclassification analysis showed that the main improvement was the decrease of false-positive results and that non-metastatic patients were reclassified into lower BSI groups. A large number of training databases including various cancer types were effective in improving the diagnostic accuracy.

## Competing interests

LE is employed by and a shareholder of EXINI Diagnostics AB. KN has a collaborative research with FUJIFILM RI Pharma, Co. Ltd, Japan. While FUJIFILM RI Pharma is involved in the distribution of BONENAVI software in Japan, KN has no relationship relevant to the contents of this study to disclose.

## Authors’ contributions

KN drafted the manuscript and LE and HH edited it. All the authors participated in the study design, and all but LE were involved in the final diagnosis and interpretation of bone scans. KN performed the statistical analysis of the data and YN confirmed it. All authors read and approved the final manuscript.
